# Fecal Microbiota Transplants for Inflammatory Bowel Disease Treatment: Synthetic- and Engineered Communities-Based Microbiota Transplants Are the Future

**DOI:** 10.1155/2022/9999925

**Published:** 2022-01-31

**Authors:** Raees Khan, Nazish Roy, Hussain Ali, Muhammad Naeem

**Affiliations:** ^1^Department of Biological Sciences, National University of Medical Sciences, Rawalpindi 46000, Pakistan; ^2^School of Life Sciences, Forman Christian College (A Chartered University), Lahore 54600, Pakistan; ^3^Department of Pharmacy, Quaid-I-Azam University, Islamabad 45320, Pakistan

## Abstract

The human intestine harbors a huge number of diverse microorganisms where a variety of complex interactions take place between the microbes as well as the host and gut microbiota. Significant long-term variations in the gut microbiota (dysbiosis) have been associated with a variety of health conditions including inflammatory bowel disease (IBD). Conventional fecal microbiota transplantations (FMTs) have been utilized to treat IBD and have been proved promising. However, various limitations such as transient results, pathogen transfer, storage, and reproducibility render conventional FMT less safe and less sustainable. Defined synthetic microbial communities (SynCom) have been used to dissect the host-microbiota-associated functions using gnotobiotic animals or *in vitro* cell models. This review focuses on the potential use of SynCom in IBD and its advantages and relative safety over conventional FMT. Additionally, this review reinforces how various technological advances could be combined with SynCom to have a better understanding of the complex microbial interactions in various gut inflammatory diseases including IBD. Some technological advances including the availability of a gut-on-a-chip system, intestinal organoids, ex vivo intestinal cultures, AI-based refining of the microbiome structural and functional data, and multiomic approaches may help in making more practical *in vitro* models of the human host. Additionally, an increase in the cultured diversity from gut microbiota and the availability of their genomic information would further make the design and utilization of SynCom more feasible. Taken together, the combined use of the available knowledge of the gut microbiota in health and disease and recent technological advances and the development of defined SynCom seem to be a promising, safe, and sustainable alternative to conventional FMT in treating IBD.

## 1. Introduction

The intestinal tract of the majority of animals including human beings is colonized by complex microbial communities since birth, called the microbiome. The composition of the microbiome differs between individuals, shows unique spatiotemporal organizations, and has a significant role in the host health and disease [1]. Thanks to the recent scientific and technological advances, particularly the discovery of high-throughput sequencing techniques, significant progress has been achieved to decipher the structure and function of the gut microbiome. The major players of the gut microbiota include Firmicutes, Actinobacteria, Bacteroidetes, Proteobacteria, and Verrucomicrobia [2]. However, we are unable to culture 99% of the microbial majority of the gut microbiota and are thus unable to explore the characteristics of all the individual microbes of the community [1]. Again, the advances in sequencing technologies have enabled us to get insights into important structure-driven functional information of the intestinal microbiome, and analysis of the microbiome of thousands of individuals revealed that each individual has a unique microbiome [2, 3]. Further, long-term dynamic studies (up to 10 years) of the microbiota of healthy human individuals revealed that the microbiome composition of the gut remains relatively stable (for up to 10 years) as compared to other parts of the body including skin and oral cavity [2, 4]. Moreover, other long-term studies revealed that a variety of biotic and abiotic factors influence the composition of the gut microbiome including dietary intake, the use of antibiotics, intestinal transit, and lifestyles [5, 6].

**Figure 1:**
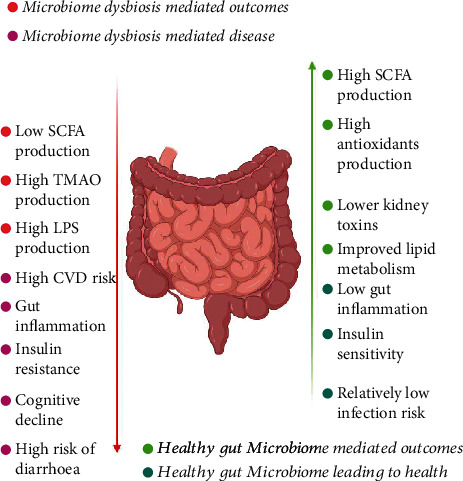
Schematic representation of the gut microbiota-mediated outcomes and their role in health and disease.

The recent technological advances and utilization of a variety of accurate approaches have further made it possible to monitor and accurately analyze the intestinal microbial community composition and how the functionality and structure of the microbiome vary in healthy and diseased individuals. This has led to remarkable success in correlating the microbiome in health and disease. Currently, the host intestinal microbiome has been associated with a variety of diseases, including various inflammatory diseases of the intestine, cancer, and obesity which are extensively reviewed recently [7]. Other medical conditions which have been correlated with lower gut microbial diversity include atopic eczema [8], type 1 and type 2 diabetes [9, 10], psoriatic arthritis [11], coeliac disease [12], arterial stiffness [13], and Crohn's disease [14]. The host-microbiome dysbiosis and associated health effects are depicted in Figure 1.

There is a gigantic amount of literature supporting the role of microbiota in health and disease. However, various ethical, medical, and microbiological concerns make it hard to establish causal relations between the microbiota and host. The use of fecal microbiome transplantations (FMTs) is an exception though, in which the intestinal microbiota of the healthy donor is transferred to a diseased individual (recipient). FMT-based interventions in humans recently caught the attention of the scientific community. This is because FMT has shown promising results in treating a variety of diseases, indicating that the intestinal microbiota does play a role in influencing the physiology and health of the host.

Following the success of FMT in treating ulcerative colitis (UC) and recurrent CDI patients over a prolonged period of 56 years, FMT-based treatments are now making their way to target several other diseases. And it all started with an important case to mention, the one reported in 1989 by Dr. Justin D Bennet [15], who suffered himself from a severe UC, and when nothing else worked for the treatment, he was finally cured by FMT from a healthy donor. Since then, FMT has been successfully employed as a potential treatment in a variety of diseases including CDI, UC, IBD, irritable bowel syndrome (IBS), chronic fatigue syndrome [16], and multiple sclerosis. FMT has been employed in more than 500 cases of chronic or recurrent CDI, resulting in an average of 95% recovery of the patients. Thus, currently, FMT is indicated only to treat recurrent CDI [17–19]. However, several clinical trials are in progress to investigate the effectiveness of FMT-based therapy in a variety of other conditions, indicating that this therapy may potentially become a novel cure option for a variety of health conditions. However, various limitations associated with conventional FMT usually end up in transient results or infections. This review focuses on the potential application of FMT in treating IBD as well as various challenges and limitations associated with conventional FMT therapies. This review also reinforces the use of defined synthetic communities (SynCom) to overcome the limitations of conventional FMT and how SynCom approaches could be combined with recent technological advances for more practical use.

## 2. Inflammatory Bowel Disease (IBD), Pathogenesis, and Drugs-Based Current Treatment Options

Inflammatory bowel disease (IBD) is a worldwide disease in the 21st century [20]. It is a chronic inflammatory disorder of the gastrointestinal (GI) tract with unknown etiology. IBD is divided into two major subtypes: Crohn's disease (CD) and ulcerative colitis (UC). UC classically involves the rectum and may affect the entire colon or part of the colon in a continuous pattern. The inflammation in UC is confined to the mucosal layer. Conversely, CD can affect any part of the GI tract but most commonly involves the ileum and perianal regions in a noncontiguous pattern, causing transmural inflammation [21]. The main symptoms of IBD include diarrhea, rectal bleeding, anorexia, and weight loss that can result in continuous bowel damage with increased risks of hospitalizations, surgeries, and colorectal cancer [22]. Children developing IBD usually have more severe diseases than adults [23]. Collectively, these conditions can result in unbearable physical and psychosocial symptoms for patients and affect society through the loss of schooling, jobs, and health care costs [24].

The exact cause of IBD is unknown. However, an inappropriate mucosal immune response against millions of antigens from food, environment, and microbiome in a genetically and/or immunologically predisposed host is believed to play a role in the pathogenesis of IBD [25]. Mucosal immune system cells such as intestinal epithelial cells, innate lymphoid cells, cells of the innate and adaptive immune system, and their secreted mediators are associated with the pathogenesis of IBD. Overall, this dysregulation in the immune system stimulates an inflammatory cascade by producing proinflammatory cytokines leading to chronic intestinal inflammation, also known as mucosal damage [26].

IBD is a public health challenge worldwide for the health professional to treat. Patients with IBD can experience symptoms at a young age [27], necessitating long-term and often costly treatment throughout their lifetimes [28]. To date, there is no cure for IBD. The aim of drug therapy is to achieve and maintain remission from inflammatory episodes. The treatment regimen of IBD consists of anti-inflammatory agents such as 5-aminosalicylates (5-ASAs), corticosteroids, immunosuppressants, and biologic agents such as tumor necrosis factor-alpha (TNF-*α*) antagonists, anti-interleukins, and anti-integrins. These drugs can induce and maintain remission from inflammatory episodes; however, they can cause serious side effects including increased risk of infections and certain cancers [29]. This may prevent IBD patients from continuing therapy and can lead to failure of therapy and increase patient morbidity and health care costs. Therefore, an utmost need is existing to find an alternative, safe, and effective therapeutic strategy for IBD therapy.

## 3. Fecal Microbiota Transplantations in Inflammatory Bowel Disease

There is a shred of growing evidence backing the gut microbiome's role in IBD pathogenesis [30]. Diversion of the fecal stream is usually utilized to treat IBD; however, a reexposure to luminal contents and reversal of the fecal stream can lead to a relapse of the disease. Additionally, antibiotics are another choice of induction therapy in IBD; also, remission can be achieved by strict enteral nutrition in CD [31, 32]. Dysbiosis is an established fact in IBD, and it has been known that the gut of IBD patients shows a relatively lower bacterial diversity particularly the loss of anaerobic bacteria [33]. Thus, gut microbiota can be targeted for novel treatment options. Initial reports regarding the use of probiotics did not result in any significant outcomes in IBD treatment [30]. A search on NIH clinical trials.gov (https://clinicaltrials.gov/) was conducted using various search terms such as fecal microbiome transplant, *Clostridium difficile* infection, Ulcerative colitis, IBD, and microbiota transplants to extract information related to trials utilizing FMT in these gut health conditions. A total of 78 clinical trials were enlisted, which were investigating the efficacy of FMT in CD or UC. Among these, 22 trials are completed and only four studies are with results whereas two studies were terminated based on the interim analysis results [34, 35]. These four completed randomized controlled trials (RCTs) revealed promising results in a small subset of UC patients (Table 1) [34–37]. Two of the terminated studies though did not show any significant difference over placebo; still, the FMT treatment outcomes appeared relatively better [34, 35]. In one study, the efficacy of FMT was dependent on donors, and the microbiota profiling of the donors resembled that of the patients who achieved remission after FMT [34]. Moreover, some other single group assignment (SGA) clinical trials also showed that FMT could result in a positive outcome in IBD (Table 1). A recent meta-analysis indicates that FMT-based interventions significantly impact remission than placebo (95% CI 2.196-5.240, *P* < 0.001). However, RCTs are lacking for CD, and various uncontrolled cohort studies with small sample sizes have revealed mixed results. For instance, using meta-analysis, a 52% remission rate was reported among 71 CD patients who received FMT [38]. However, among the studies pooled into the meta-analysis, only a single one was a large cohort study and the remission rate was attributed mainly to it [39]. Furthermore, no endoscopic remission was observed eight weeks post-FMT in CD patients [40]. Because the FMT outcomes in IBD patients are not constant, this treatment option should still be considered an experimental one. More studies are needed regarding suitable donor selection, selection of highly responsive patients, and processing of feces under anaerobic conditions. Moreover, we still do not know what should be the proper timing for FMT interventions in IBD patients. Should FMT be used as the primary treatment or should be applied postinduction therapy? The good thing is that various trials that are currently ongoing may help to address the above questions. Additionally, this will further pave the way for using FMT as a potential treatment option in the future for IBD patients. FMT is also utilized in IBD patients who experience recurrent *Clostridium difficile* infection (rCDI), and a meta-analysis revealed that FMT could result in significant outcomes for treating rCDI in IBD patients (initial cure rate of 81%) compared to non-IBD patients [41]. Additionally, FMT has been known equally significant to treat rCDI both in CD and UC patients. Some of the reported adverse outcomes of FMT include IBD flare; however, it is still debatable whether this flare was associated with FMT intervention or was the result of CDI.

## 4. Challenges and Limitations Associated with Conventional Fecal Microbiota Transplantation

**Figure 2:**
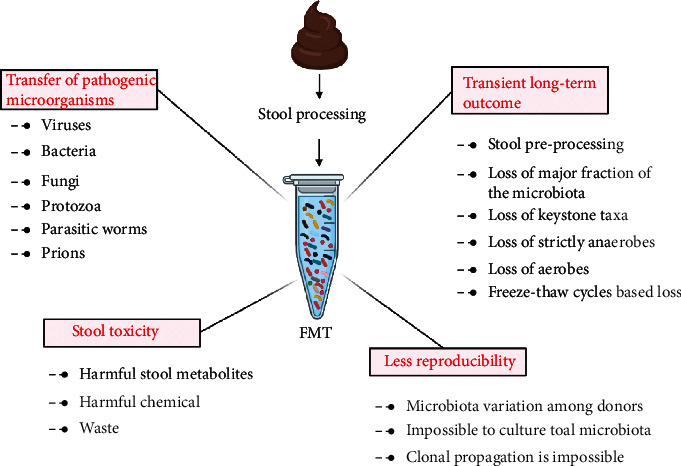
Disadvantages and limitations of conventional FMT. Various disadvantages include pathogen transfer, transient results, stool toxicity, and difficulty in reproduction.

FMT-based therapies seem to be a promising treatment option for a variety of diseases including IBD; however, it has a variety of limitations resulting in transient and adverse outcomes (Figure 2). One of the major limitations is long-term safety. Although FMT is considered “safe” or “natural” or even “organic” by a majority of the recipients and practitioners, it can potentially be harmful and risky. There is a potential risk that the fecal material from a healthy donor may expose the patient to enteric pathogenic microorganisms and thus spreading and contracting the disease. In a recent study, two patients who recently received FMT were reported being infected with extended-spectrum beta-lactamase (ESBL)-producing pathogenic *Escherichia coli* bacteremia. The source of infection in both patients was tracked back to the same donor stool. One of the two patients expired [42]. FMT-mediated infections have been reported in other cases as well, where the source of infection was supposed to have been presented by the fecal microbiome [43–45]. This warrants the need for improved donor screening to minimize the risk prior to FMT-based therapies.

Another important concern is the reproducibility and sustainable long-term use of FMT for a stable outcome. Though FMT shows promising results in the case of CDI, in other diseases such as IBD, it usually ends in the transient outcome. This indicates the complexity of the host-microbiome interactions and the low-key technology and poor practices leading to the loss of the major fraction of the original microbiota. Since each individual carries a unique and stable microbiota, it becomes very important to identify a healthy donor microbiome and ensure the reproducibility of the exact replica of that microbiome for long-term sustainable use and stable clinical outcome. Several factors lead to the loss of a major fraction of the fecal microbiota and thus transient results. For instance, majority of the intestinal microbes are strictly anaerobes, and fecal samples are mostly processed under aerobic conditions, which will instantly kill the anaerobes [46]. On the other hand, if handled anaerobically, the strictly aerobes will vanish. Also, the routinely used storage techniques at low temperatures (-20°C to -80°C) have been known to lead to significant loss of community members of the original microbiota, as a result of the freeze-thaw cycles [47]. This can further result in instability of the clinical outcome in FMT therapy. Moreover, knowledge is lacking for the long-term freeze-based storage (~10 years) of the intestinal fecal materials and their efficacy. Also, stool preprocessing for FMT preparations can lead to significant loss or damage to the major fraction of the microbial community resulting in the loss of approximately 50% of members [48]. Furthermore, cultured-based approaches also seem not suitable, because the gut microbiome is known to be composed of more than 2000 different species majority of which (≥90-99%) cannot be cultured. Also, the stool material itself consists of a variety of harmful chemicals, metabolites, and waste which can pose potential harm to the donor. In summary, there is a large disparity between the currently used technology for FMT-based treatment and the delicate knowledge of the gut microbiota. Therefore, ensuring the long-term safety of the donors in FMT-based treatments should be the primary priority. Also, the production of a reproducible functional microbiome from a single healthy donor may ensure long-term sustainable use and stable outcomes.

## 5. Synthetic and Engineered Microbial Communities to Understand Microbiota-Assisted Functions

**Figure 3:**
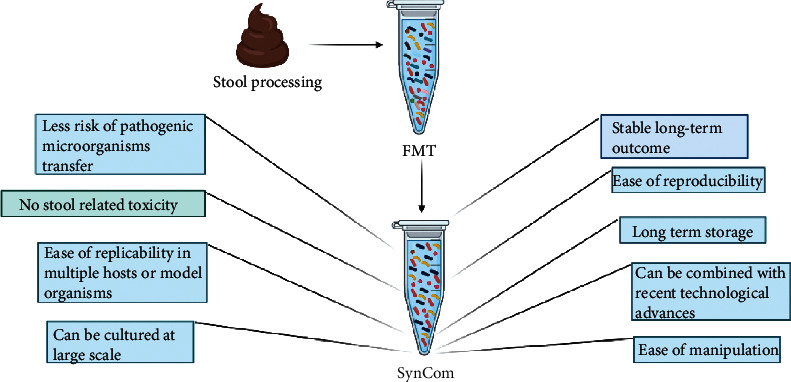
Advantages of SynCom-based transplants over conventional FMT. Various advantages of the SynCom-based FMT include relatively more safety, stable results, relatively less stool toxicity, and ease of reproducibility.

The human gut harbors a diverse array of microorganisms, and it is quite challenging to assess how individual microbes interact with the host and to understand microbiome-mediated functions. Culture-based approaches have been used to decipher host-microbiome interactions; however, various limitations such as only a minute fraction of the total gut microbiota being culturable make these approaches difficult to understand host-microbiome interactions [49–53]. This indicates that conventional screening approaches are not ideal to decipher the host-microbiome complexity and microbiome-assisted functions. A possible solution to this problem is the concept of synthetic bacterial community (SynCom), which is a structurally defined/controlled community. SynCom consists of relatively few known cultured microbial members, and it acts as a representative of the original host-microbiome functions and structure [53]. The SynCom approach has a great advantage in that we can manipulate this community by simply adding, eliminating, or substituting one or a few strains to achieve desired functions including probiotic properties and disease remission (Figure 3). Additionally, such manipulations can even be introduced at the strain genetic levels as well; for instance, individual functions of the SynCom member microbes can be deleted or improved using gene silencing or increased expression, respectively. Because the SynCom microbial members are culturable, this renders the member strains suitable for dissecting the structural complexity and microbiota-associated functions via reductionist approaches. SynCom approaches could be of great use while testing germ-free organisms to decipher the quantitative and qualitative traits of the host driven by the host-associated microbiota. Moreover, the use of SynCom has become an important and practical alternative to the use of conventional FMT as it lacks most of the limitations associated with conventional FMT. These include sustainable use, stable outcome, ease of reproducibility, and long-term safety [53].

The SynCom approach has been widely utilized to determine its safety and functionality in various pathological conditions [54]. Though the source organisms used in these SynCom were derived mostly from humans, none of these were tested back in humans and alternative hosts used were either germ-free mice, rats, or pigs [54]. Next-generation sequencing- (NGS-) based metagenomic studies have been utilized to explore the gut microbial community structures of humans and other animals both in healthy and diseased conditions. Further insights into such NGS data such as relative abundance analysis and network analysis have shed light on microbial dynamics as well as member strains which are crucial for maintaining the structure and function of the microbial community. Moreover, recent advances in culturomics have led to an increased number of cultured organisms from the gut, particularly those which were previously considered unculturable [55]. For instance, various combinations of the long-known popular Altered Schaedler Flora- (ASF-) based SynCom have been utilized in different organisms to see its applicability in improving various pathological conditions (extensively reviewed by [54]). One study used ASF-based SynCom (comprised of 8 different strains) in mice to see how it affects the death rate after *C. botulinum* infection, fecal *C. botulinum* toxin excretion, and colonization pattern [56]. Results revealed that SynCom though did not prevent infection; however, the death rates were significantly lower in mice who received SynCom-based transplantations compared to the nontreated controls. Such functional studies thus indicate that SynCom-based approaches would be a powerful technique to dissect host-microbiome interactions. The major drawback observed for the ASF communities was that it poorly represents the dominant flora of the gut. Hence, SynCom was further modified to include members representative of the dominant flora as well. Thus, a variety of formulations were tested such as the Oligo-MM (murine microbiota) which consisted of twelve members. The Oligo-MM-based SynCom revealed that this community could provide significant resistance against S*almonella enterica serovar Typhimurium* colonization and was even relatively better than ASF-based communities [57]. So far, only a single study has been conducted assessing the significance of SynCom in IBD in mice. The role of pathogenic bacteria *Helicobacter hepaticus* in the presence of normal ASF flora was determined in IBD [58]. This flora consisted of eight anaerobic species. Results revealed that even the presence of a single pathogen could lead to IBD conditions in the presence of normal representative flora. This was the pioneering study which revealed that the gut flora has a role in establishing IBD condition.

Though SynCom has a broad range of practical applications in decoding the functional prospects of host-microbiota, still it is unclear whether the SynCom-based outcomes observed in most of the animal models used could be replicated in humans and closely related other hosts as well. The fraction of the organisms cultured so far from the human gut is so small relative to the total microbial diversity of the gut. Therefore, there is a dire need to cultivate more organisms from the gut. Particular emphasis should be on culturing organisms that are abundant in the gut but are still not cultured or with very few cultured members. One such example is the Verrucomicrobia phyla, which are usually present in abundance in the human gut, but so far the cultured members are limited to few representative strains. Once we have enough number of cultured representatives of the gut microbiome, only then will we be able to design better SynCom which could then be of more practical use in human hosts.

## 6. Perspectives: How SynCom Could Be of Better Use in IBD and Address the Conventional FMT Limitations

The recent technological advances in the field of multiomics including but not limited to structural and functional metagenomics, metatranscriptomics, metaproteomics, metabolomics, and the ease of big data analysis have been largely utilized to elucidate the structure and function of the host-associated microbiota [59]. Few big projects in the fields to mention that have greatly facilitated the understanding and future goals include the Human Microbiome Project, the American Gut, the European microbiome project, and the Asian microbiome project. This has further facilitated the provision of gut bacterial strain banks comprising of diverse isolates as well as has standardized the host-associated microbiota structural and functional profiling protocols [18, 60].

Recently, the concept of the core microbes has been introduced, suggesting that certain bacterial groups are critical for maintaining the structure and function of the gut microbial community [61–63]. However, there is a need for suitable model systems to decipher and test the role of these core microbes and to establish causality in terms of microbiota-assisted functions in animal models. Such model systems will enhance our understanding of the host-microbiota interactions and also interactions among the diverse members within the microbial community. The existence of inherent complex interactions among microbial community members makes it difficult to understand and assign the resultant phenotype to an individual microbe or to a subgroup of that particular microbial community. Such complex interactions should be investigated with the help of suitable model systems. Utilizing culture-based approaches to culture diverse groups of organisms from the gut further decreases the fickleness as a result of the complexity of the microbial community. Further, such approaches make it feasible to test principles of interactions among the host and its associated microbiota as well as intrinsic interactions among the microbial community members under controlled conditions. There is a dire need for an advanced strategy to dissect the host-microbiome interactions at the gut interface in various gastrointestinal conditions such as IBD. This can further elucidate the individual role of both sides (the gut microbiota and the host immune response) in IBD.

SynCom comprising of several culturable bacterial isolates of the human gut could be one such alternative to address the limitations of conventionally used FMT as well as those posed by the inherent complexity of the gut microbiota. The use of SynCom may shed light on how the dynamics of gut microbial community composition contribute to IBD development in terms of intermicrobial interactions: physical, chemical, and genetic interactions. Moreover, the use of SynCom in model organisms would further explain the underlying functional mechanisms and intermicrobial as well host-microbiota interactions leading to disease or health. Therefore, defined SynCom stand as the only promising validation tool for host-microbiota-associated function dissection *in vivo*. Also, this approach allows one to test and transfer the outcomes in the laboratory, and the output can ultimately be translated and utilized at a broader scale such as the treatment of various gastrointestinal conditions including IBD via SynCom-based transplants.

A variety of clinical trials have assessed the efficacy of conventional FMT in various gastrointestinal disorders including IBD (Table 1). Moreover, various conventionally used combinations of cultured microbes have been utilized in model organisms including mice, rats, and pigs to see if they can lead to a significant outcome [58, 64]. However, most of the studies have neglected postconventional FMT gut microbiota analysis. A significant number of studies are required to investigate the positive and negative outcomes post-FMT transfer and the associated microbial community structure. Comparative analysis of the microbial communities in both cases (positive and negative outcomes) could shed light on which microbial taxa, in particular, are responsible for leading to IBD or curing the IBD. Additionally, the big data coming out from such projects are complex, and such complexity of the microbial community structure and dynamics could potentially be solved by the recent technological advancements in the field of artificial intelligence (AI), including machine learning algorithms that could integrate huge metagenomic and microbiome data [65, 66]. Luckily, a gigantic amount of such comprehensive data has been sufficiently generated in the field of medical sciences. Therefore, AI-based big data comparative analysis of the gut microbiota in normal individuals, in individuals with IBD, and in individuals who have received FMT post-IBD could further enhance our understanding of the intermicrobial interactions, host-microbiome interactions, elucidating the role of the gut microbiota leading to IBD or curing IBD and finally fine-tuning of SynCom for future medical applications.

Various human microbiome-related studies have explored the microbial community structure that lives in association within the human gut [5]. Additionally, the presence of multipartite interactions (host-microbiota interactions and microbe-microbe interactions within the community) have been explored to some extent; however, much is still unknown and various important questions still do exist, and answering those will help in the design and utilization of SynCom for more practical use such as in conditions like IBD. Ultimately, there are still several important questions to be answered. The following are some of those questions: (1) What are the underlying mechanisms that gate and maintain a unique gut microbiota structure and lead to a healthy gut? (2) Is it the human host immune system acting as a gatekeeping system selectively allowing some (but not all) microbes to colonize the gut? (3) Have the gut colonizers evolved specific mechanisms to bypass the host gatekeeping system? (4) Is the differentiation among pathogenic and commensals driven by host genetic factors? (5) What factors (other than genetic) are involved which help hosts in recognizing, nurturing friend microbes, and maintaining a healthy gut? (6) How does the gut microbiota modulate host functions? Is it the dysbiosis of the gut microbiota that lead to disease conditions such as IBD? Or is dysbiosis of the gut microbiota the result of IBD itself? Do the gut microbiota and the host immune system work together in maintaining a healthy gut; if yes, then who does what and to what extent? Much is still waiting answers. There is a dire need to combine the systematic and reductionist approaches to dissect the individual roles of host and associated microbiota in conditions like IBD. For instance, intestinal cell line and stem cells, intestinal organoids (based monocultures, transwell, gut on a chip), ex vivo intestinal cultures, AI-based refining of the microbiome structural and functional data, multiomic approaches, and SynCom approaches could be combined to dissect the microbiota-assisted functions and the role of gut microbiota in various gut diseases such as IBD. Such complementary translational research would not only enhance our understanding of the complex interactions between host and associated gut microbes but also be practically applied to design better SynCom, a safe and sustainable alternative to conventional FMT, and to achieve more controlled and robust treatment of gut inflammatory disorders such as IBD.

## Figures and Tables

**Table 1:** Completed clinical trials investigating FMT as a potential therapy for IBD.

Feature of study	Completed RCT trials				Completed SGA trials			
	Moayyedi et al.	Rossen et al.	Paramsothy et al.	Costello et al.	NCT02108821	NCT03106844	NCT01560819	NCT02049502
Study design	Double-blind RCT	Double-blind RCT	RCT	RCT	Single group assignment	Single group assignment	Single group assignment	Single group assignment
Number of patients (placebo)	75 (37)	48 (25)	81 (40)	73 (35)	23 (NA)	50 (NA)	9 (NA)	8 (NA)
Treatment regimen	6 FMTs	2 FMTs	40 FMTs	3 FMTs	1 FMT	1 FMT	20 FMT	Single FMT
Comparator (placebo)	Water	Autologous FMT	Water	Autologous FMT	None	None	None	None
Route of administration	Lower GI, enema	Upper GI, duodenal tube	Lower GI, retention	Lower GI, retention	Upper GI, jejunal intubation	Lower GI, colonoscopy	Retention enema	Lower GI, sigmoidoscopy
Stool donor per suspension	Single donor	Single donor	Multiple donors	Multiple donors	NA	NA	Multiple donors	Single donor
Follow-up	6 weeks	12 weeks	8 weeks	8 weeks	26 weeks	8 weeks	4 weeks	13 weeks
Primary endpoint	Endoscopic remission	Endoscopic remission	Endoscopic response	Endoscopic remission	Occurrences of adverse events	Recurrence of CDI in IBD patients	Improvement in PUCAI score	Improvement of pouchitis symptoms based on mPDAI
Primary outcome FMT versus comparator	24% (9/38) versus 5% (2/37) *P* = 0.03	30% (7/23) versus 20% (5/25) *P* = 0.51	27% (11/41) versus 8% (3/40) *P* = 0.02	32% (12/38) versus 9% (3/35) *P* < 0.01	52.17% (12/23)	8.2% (4/49)	Improvement in PUCAI score in all patients; 100% (9/9)	Improvement in mPDAI score in all patients; 100% (9/9)

Abbreviations: FMT: fecal microbiota transplant; GI: gastrointestinal; RCT: randomized controlled trial; UC: ulcerative colitis; mPDAI: modified pouchitis disease activity index; PUCAI: Pediatric Ulcerative Colitis Activity Index; SGA: single group assignment.
